# Annual Report of the Mortality of the City of Buffalo, for the Year 1856

**Published:** 1857-04

**Authors:** 


### EDITORIAL DEPARTMENT.

*Annual Report of the Mortality of the City of Buffalo, for the Year* 1856.—In a neat pamphlet the Common Council has published the mortality statistics of the city for the past year, prepared by Dr. C. L. Dayton, the Health Physician. This document officially emanates from the Board of Health, and contains also a summary of the expenses of this department of our municipal government.

Dr. Dayton has given us a report exceedingly creditable to himself. His tables are worked out with much care, and are fully as elaborate as they can be made with the amount of data at his command. As this is the third report only which the city has published, he had but few materials with which to institute comparisons. His report differs from the two previous ones in that he has classified his diseases, making them more convenient for the purposes of study, a course which we trust will be adopted in future.

The year was free from any epidemics, and the evidence which is here furnished of the healthfulness of our city is a subject of congratulation for our citizens; and after the emotions of thankfulness which such a knowledge should inspire, they may be pardoned in referring to it as an element in the present and prospective growth and prosperity of the town. The health of our city is a subject which our citizens often refer to, in terms of pride, and the figures of this report will amply sustain them in their boastings.

But we design to refer more particularly to the report, and allow the figures to speak for themselves. The total number of deaths, from all causes, during the year 1856, as reported, is 1,802, of which 70 were still born, and should be deducted in the estimate of the influences of diseased actions; and 131 deaths are chargeable to external causes, which includes accidents of all kinds, intemperance, murder, poison and suicides. After we have made these deductions, we have but 1601 deaths really the result of diseases.

If we institute a comparison with the two previous years, we find there was

In 1854, a cholera year, and after deducting 572 deaths from that disease, a mortality from all other causes, of - - - 2,364
In 1855, the number of deaths, after deducting 11 deaths from cholera, was - - - - - - - - - - 1,845
In 1856, the total mortality from all causes was - - - - 1,802

The constant preponderance toward a standard of mortality, so uniform that it may be taken as a healthy standard, will not escape notice. The depressing influence upon the vital powers, and the inability to withstand the attacks of ordinary diseases, during the prevalence of an epidemic of cholera, will not fail to attract attention.

During the year 1856, 501 deaths, or 27.802 per cent. of deaths from all causes, were from zymotic diseases. After we make the deduction of still born, and deaths from external causes, we find that actually 31.29 per cent., or nearly one-third of all the deaths from actual disease, were from the class of zymotics. For the convenience of comparison with previous years, we present the followii g table:

| | Total Deaths. | By Zymotics. | Per cent. |
|---|---|---|---|
| In 1854, - - | 2,936 | 1,321 | 44.99 |
| In 1855, - - | 1,856 | 559 | 30.11 |
| In 1856, - - | 1,802 | 501 | 27.80 |

This should arrest attention. The comparative influence of zymotics in the production of deaths, has been elaborated by Dr. Newman, in his report on the "Sanitary Police of Cities," made to the American Medical Association. A most important lesson is taught upon this subject. Those diseases most under the control of man, in their modes of increase and propagation, are permitted to become the most fatal of all the diseases which afflict the human body.

The proportion borne by the two sexes to the whole mortality is as follows:

| | |
|---|---|
| Males, - - - - - - | 989 |
| Females, - - - - - | 732 |
| Sex not stated in the certificates, - | 81 |

Table III, of the report, gives the ages of the deceased, arranged so as to exhibit the mortality from month to month. The immense mortality of infantile life as there displayed, is a subject worthy more than a passing notice. Of the entire number of deaths from all causes 957, or 53.107 per cent.

were under three years of age! Between the ages of three and five years, there were only 50 deaths. The first thirty days of infantile life are the most fatal, 132 deaths occurring during this period.

This subject as illustrative of the comparative chances of life and of the powers of the human system to ward off the attacks of disease, will warrant us to insert all the figures of the table bearing upon this point, viz:

| | |
|---|---|
| Still-born, | 74 |
| 1 day, | 27 |
| Between 1 and 30 days, | 105 |
| Between 1 and 6 months, | 221 |
| Between 6 and 12 months, | 222 |
| Between 1 and 3 years, | 308 |
| Between 3 and 5 years, | 50 |
| Between 5 and 10 years, | 58 |
| Between 10 and 20 years, | 72 |
| Between 20 and 30 years, | 174 |
| Between 30 and 40 years, | 156 |
| Between 40 and 50 years, | 116 |
| Between 50 and 60 years, | 74 |
| Between 60 and 70 years, | 57 |
| Between 70 and 80 years, | 28 |
| Between 80 and 90 years, | 9 |
| Between 90 and 100 years, | 4 |
| 100 years and upward, | 0 |
| Total, | 1755 |

Ages not given in the certificates of 47 deaths.

Upon the subject of the nativities of those dying, the figures are less definite than could be desired, but it is almost impossible, we presume, to obtain full facts relative to the birthplace of all the parties. In 255 of the certificates of death, no country is returned. So far as given in the certificates, the nativities of the deceased, are United States, (white and colored,) 790. Foreign, 757. Those born in the United States, of foreign parents, are included under the head of natives of the United States.

Table VI gives a nosological classification of the causes of death, with the seasons of the year in which they occur, and which we give entire:

| SEASON OF DECEASE, WITH CAUSES. | Spring. | Summer. | Autumn. | Winter. | Total. |
|---|---|---|---|---|---|
| I. Zymotic Diseases, | 41 | 228 | 149 | 83 | 501 |
| II. Uncertain or Variable Seat, | 22 | 29 | 50 | 34 | 135 |
| III. Brain and Nervous System, | 80 | 81 | 59 | 71 | 291 |
| IV. Respiratory Organs, | 83 | 49 | 77 | 76 | 285 |
| V. Organs of Circulation, | 4 | 7 | 4 | 5 | 20 |
| VI. Digestive Organs, | 18 | 24 | 33 | 23 | 98 |
| VII. Urinary Organs, | 1 | 1 | 1 | 2 | 5 |
| VIII. Generative Organs and Childbirth, | 4 | 3 | 1 | 4 | 12 |
| IX. Locomotive Organs, | 2 | 2 | 3 | 2 | 9 |
| X. Diseases of the Skin, | 0 | 0 | 0 | 0 | 00 |
| XI. Old Age, | 11 | 12 | 13 | 10 | 46 |
| XII. External Causes, | 21 | 49 | 47 | 14 | 131 |
| XIII. Still-born, | 19 | 19 | 20 | 12 | 70 |
| XIV. Unknown, | 41 | 59 | 54 | 45 | 199 |
| Totals, | 347 | 563 | 511 | 381 | 1802 |

The following comparison is drawn between the mortality in the city for the three years past, from cholera and consumption:

| | | | | |
|---|---|---|---|---|
| 1854, | Deaths from cholera, | 572 | Deaths from consumption, | 266 |
| 1855, | " " " | 11 | " " " | 250 |
| 1856, | " " " | 00 | " " " | 198 |
| | Total, - - | 583 | Total, - - | 714 |

The subject is made the text for some judicious remarks upon the hygenic causes which enter into the development of phthisis.

Only eight cases of small-pox were reported to the Board of Health during the year, and but one of these was fatal.

The report contains some valuable suggestions relative to the sanitary improvement of our city, which we must omit for want of room, and refer to the report itself, and we trust we have given enough to convince our readers that the paper is not unworthy of their careful study. We will close the subject, by urging upon our medical men the importance of their giving all the aid in their power, to render future reports still more accurate in the details than the present one, meritorious as it is.

